# Insights to ligand binding to the monoamine transporters—from homology modeling to LeuBAT and dDAT

**DOI:** 10.3389/fphar.2015.00208

**Published:** 2015-09-24

**Authors:** Heidi Koldsø, Julie Grouleff, Birgit Schiøtt

**Affiliations:** ^1^Department of Biochemistry, University of Oxford, Oxford, UK; ^2^inSPIN and iNANO Centers, Department of Chemistry, Aarhus University, Aarhus C, Denmark

**Keywords:** leucine transporters, dopamine transporter, serotonin transporter, norepinephrine transporter, LeuBAT, antidepressant, psychostimulants

## Abstract

Understanding of drug binding to the human biogenic amine transporters (BATs) is essential to explain the mechanism of action of these pharmaceuticals but more importantly to be able to develop new and improved compounds to be used in the treatment of depression or drug addiction. Until recently no high resolution structure was available of the BATs and homology modeling was a necessity. Various studies have revealed experimentally validated binding modes of numerous ligands to the BATs using homology modeling. Here we examine and discuss the similarities between the binding models of substrates, antidepressants, psychostimulants, and mazindol in homology models of the human BATs and the recently published crystal structures of the *Drosophila* dopamine transporter and the engineered protein, LeuBAT. The comparison reveals that careful computational modeling combined with experimental data can be utilized to predict binding of molecules to proteins that agree very well with crystal structures.

## Introduction

The human biogenic amine transporters (BATs) represent important drug targets for the treatment of many psychiatric diseases such as depression, anxiety, obesity, drug abuse, obsessive compulsive disorder, attention deficit hyperactive disorder, and schizophrenia (Jimerson et al., 1997; Hahn and Blakely, 2002; Gainetdinov and Caron, 2003; Murphy et al., 2004; Delorme et al., 2005; Sutcliffe et al., 2005; Mazei-Robison and Blakely, 2006; Garfield and Heisler, 2009). They are also the target of psychostimulants such as amphetamine, cocaine, and ecstasy (Ritz et al., 1987; Rudnick and Wall, 1992; Eshleman et al., 1994). The BATs includes the serotonin (SERT), dopamine (DAT), and norepinephrine (NET) transporters, responsible for re-uptake of the neurotransmitters SERT, DAT, and NET, respectively and they function by terminating synaptic signaling (Rudnick and Clark, 1993; Reith, 2002; Kristensen et al., 2011). When blocking these proteins, the concentration of the neurotransmitter within the synapse is elevated hereby relieving the symptoms of many psychiatric diseases.

Rational design of drugs targeting the BATs has been difficult due to the lack of high resolution structural information. However, since 2005, when the first protein crystal structure of a protein belonging to this family of transporters, the bacterial leucine transporter from *Aquifex aeolicus*, LeuT, was published (Yamashita et al., 2005), homology modeling of the monoamine neurotransmitter transporters has been possible. The structure of LeuT revealed an architecture consisting of 12 transmembrane α-helixes (TMs) with both the N- and C-terminal placed intracellular and a centrally placed substrate binding site (known as S1) in the transmembrane part of the proteins, close to two sodium ion binding sites, Na1 and Na2, respectively (Yamashita et al., 2005). The structure furthermore revealed a structural repeat between TM1-TM5 and TM6-TM10 linked by a pseudo C2-rotation axis perpendicular to the membrane normal. This inverted repeat (Forrest et al., 2008) is now commonly known as the LeuT-fold and is found in a broad class of proteins, which in addition to BATs also include Mhp1 (Weyand et al., 2008; Shimamura et al., 2010), BetP (Ressl et al., 2009), CaiT (Schulze et al., 2010; Tang et al., 2010), AdiC (Fang et al., 2009), vSGLT (Faham et al., 2008), and ApcT (Shaffer et al., 2009).

Later, new crystal structures of LeuT have provided some insight into how inhibitors and antidepressants might bind to this bacterial transporter (Singh et al., 2007, 2008; Zhou et al., 2007, 2009; Quick et al., 2009). Crystal structures of LeuT co-crystallized with the inhibitor tryptophan showed two tryptophan molecules bound, one in S1 and the other in the extracellular vestibule, also known as the S2 site. In comparison to LeuT crystal structures with bound substrate, such as alanine or leucine, the Trp-LeuT complex has the protein in an outward-open conformation in which the solvent has access to the substrate molecule in S1, caused by a rotation of the two aromatic residues, otherwise guarding the substrate binding site, and a small tilt in TM4 (Singh et al., 2008). Several structures of LeuT have later confirmed the presence of the S2 site in the outer vestibule, and they show that many different types of molecules can bind here, e.g., detergents (Quick et al., 2009) and inhibitors, such as the amino acid tryptophan (Singh et al., 2008) as well as antidepressants (Singh et al., 2007; Zhou et al., 2007, 2009). The relevance of these binding models in relation to how the human BATs are being inhibited has, however, been strongly debated (Rudnick, 2007; Piscitelli et al., 2010; Quick et al., 2012).

The first high-resolution insight to drug binding to the BATs was elucidated in 2013 through the publication of crystal structures of engineered LeuT, LeuBAT (Wang et al., 2013), and the *Drosophila* DAT (dDAT; Penmatsa et al., 2013). LeuBAT is an engineered version of LeuT where the key residues within the central binding site have been mutated to resemble the pharmacology of the BATs. Twelve structures of LeuBAT with various antidepressants co-crystallized were published. The structures included LeuBAT in complex within selective serotonin reuptake inhibitors (SSRIs), serotonin-norepinephrine reuptake inhibitors (SNRIs), and tricyclic antidepressants (TCAs) with varying amount of point mutations (Δ5, Δ6, and Δ13). The crystal structures included the binding of mazindol in the Δ5 and Δ6 structures, whereas seven structures were obtained in the Δ13 LeuBAT including the binding of the TCA clomipramine (CMI; Wang et al., 2013). The dDAT structure published in 2013 contained the TCA nortriptyline within the central binding site (Penmatsa et al., 2013) in the same binding mode as found for CMI in LeuBAT (Wang et al., 2013), revealing that the previous LeuT crystal structures with co-crystallized antidepressants in the S2 site most likely do not reflect the relevant binding mode of these drugs in the BATs.

Importantly, in May 2015 an arsenal of new crystal structures of dDAT with various ligands bound were published (Penmatsa et al., 2015; Wang et al., 2015). These new crystal structures included the substrate DAT, the psychostimulants *D*-amphetamine, (+)-methamphetamine, cocaine and the cocaine analog RTI-55 as well as SNRIs, NET-specific reuptake inhibitors (NRIs) and SSRIs bound. For the first time it is accordingly possible to directly compare the binding of substrates to that of different types of inhibitors in a DAT structure.

Several models have been published describing the binding of substrates, antidepressants, psychostimulants and mazindol to either of the human BATs using homology models constructed based on the structure of the bacterial homolog LeuT (Forrest et al., 2007; Huang and Zhan, 2007; Jørgensen et al., 2007a,b; Beuming et al., 2008; Celik et al., 2008; Indarte et al., 2008; Andersen et al., 2009, 2010, 2014; Huang et al., 2009; Tavoulari et al., 2009; Combs et al., 2010; Gedeon et al., 2010; Koldsø et al., 2010, 2011, 2013a,b; Sarker et al., 2010; Sinning et al., 2010; Schlessinger et al., 2011; Shan et al., 2011; Merchant and Madura, 2012; Severinsen et al., 2012, 2014; Dahal et al., 2014; Wilson et al., 2014).

Herein, we compare the binding of drugs to dDAT (Penmatsa et al., 2013; Wang et al., 2015) and LeuBAT (Wang et al., 2013) obtained from the crystal structures to previously built homology models that have been constructed based on the bacterial homolog LeuT (Yamashita et al., 2005). The comparison clearly illustrates that it is possible to predict the binding of drugs to the human BATs through carefully calculated computer models in combination with experimental validation.

## Comparison Between dDAT, LeuBAT, and Homology Models of Human BATs

### Substrate Binding

**FIGURE 1:**
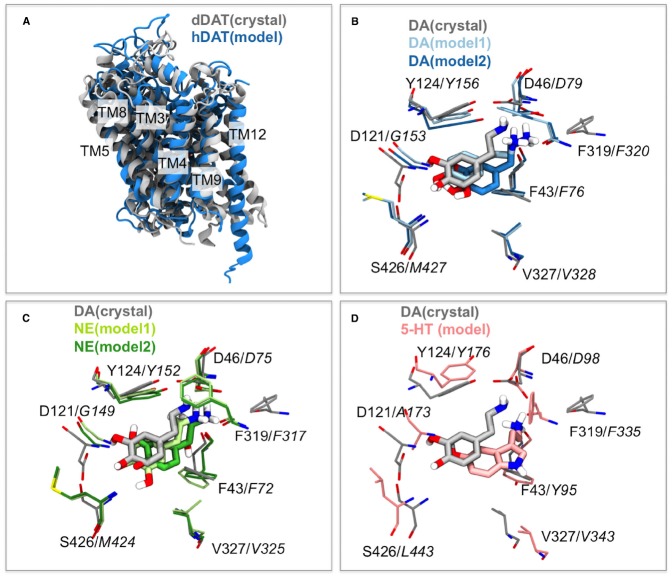
**Similarities in dDAT and hDAT structures and substrate binding. (A)** The overall structure of dDAT (Wang et al., 2015) and the homology model of hDAT (Koldsø et al., 2013b) based on an outward occluded LeuT structure are almost identical with the largest difference being in TM12 where a kink is observed within the dDAT structure. **(B)** Comparison of the binding mode of the substrate dopamine (DA) within the dDAT crystal structure (gray; Wang et al., 2015) and two binding modes obtained from modeling (Koldsø et al., 2013b) shown in light and dark blue. Italic residue numbers are from the hDAT homology model and normal labels belong to dDAT. **(C)** Comparison of the binding mode of the substrate DA within the dDAT crystal structure (gray; Wang et al., 2015) and the substrate norepinephrine (NE) in two binding modes obtained from homology model of hNET (Koldsø et al., 2013b) with the models shown in light and dark green. Italic residue numbers are from the hNET homology model. **(D)** Comparison of the binding mode of the substrate DA within the dDAT crystal structure (gray; Wang et al., 2015) and the substrate serotonin (5-HT) in the experimental validated binding mode within a homology model of hSERT (Koldsø et al., 2013b) with the model shown in pink. Italic residue numbers are from the hSERT homology model.

The structure of the dDAT protein compared to a homology model of the human DAT previously published (Koldsø et al., 2013b) is shown in Figure 1A. The general agreement between the homology model of hDAT and the crystal structure of dDAT is very good and the principal differences are observed within TM12, which is slightly kinked in the dDAT structure. One of the largest differences observed between LeuT and dDAT is also TM12 as described previously (Penmatsa et al., 2013) and the differences observed here is therefore not surprising since the hDAT model has been based on the LeuT structure, in which TM12 is not kinked.

The homology models of hDAT, hNET, and hSERT are compared with the dDAT crystal structure by alignment of the central binding site residues (Figure 1). For the alignment, residues within 5 Å of the co-crystallized nortriptyline in the dDAT structure are selected (Penmatsa et al., 2013). The residues used from dDAT are S320, F319, L321, D46, G322, A44, F43, F325, S421, V327, G425, S426, S422, A117, D121, I116, V120, Y123, Y124, A479, and the Cα atoms of the corresponding residues within the other transporters based on structural and sequence alignment (Beuming et al., 2006). The location of the DAT substrate within dDAT from the crystal structure strongly resembles the location of DAT proposed by Koldsø et al., 2013b; Figure 1B) and also proposed by others (Beuming et al., 2008; Shan et al., 2011). The position of the binding site residues is also very similar with only small deviations at a few positions. Similarly the binding of NET in hNET obtained by homology modeling (Koldsø et al., 2013b) strongly resembles the position of the very similar substrate DAT within dDAT (Figure 1C). Additionally the experimental validated orientation of SERT within hSERT (Celik et al., 2008) overlay with the position of DAT within dDAT (Figure 1D). There are accordingly excellent agreements between the substrate-bound dDAT structure and the substrate binding modes predicted based on homology modeling (Beuming et al., 2008; Celik et al., 2008; Koldsø et al., 2011, 2013b; Schlessinger et al., 2011; Shan et al., 2011).

### Psychostimulants Binding

**FIGURE 2:**
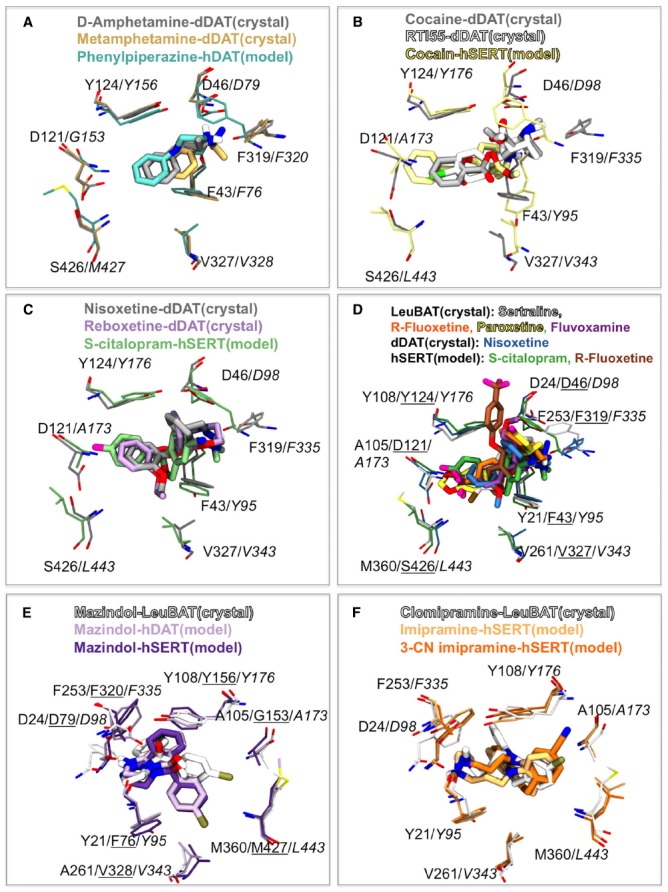
**Comparison of psychostimulants, mazindol and antidepressant binding between the dDAT and LeuBAT crystal structures and human BAT homology models. (A)** Comparison of releaser binding. *D*-amphetamine (gray) and (+)-methamphetamine (light brown) from dDAT crystal structures (Wang et al., 2015) and PP (cyan) within a hDAT homology model (Severinsen et al., 2012). hDAT labels are shown in italic. **(B)** Comparison of cocaine and analogs binding. Cocaine (light gray) and RTI-55 (white) from dDAT crystal structures (Wang et al., 2015) and cocaine (yellow) within a hSERT homology model (Koldsø et al., 2013a). hSERT labels are shown in italic. **(C,D)** Comparison of NRI and SSRI binding. **(C)** Nisoxetine (gray) and reboxetine (light purple) from dDAT crystal structures (Penmatsa et al., 2015) and *S*-citalopram (green) within a hSERT homology model (Koldsø et al., 2010). hSERT labels are shown in italic. **(D)** Sertraline (light gray), *R*-fluoxetine (orange), paroxetine (yellow), fluvoxamine (purple) within LeuBAT crystal structures (Wang et al., 2013) and nisoxetine (blue) within dDAT (Penmatsa et al., 2015) compared to *S*-citalopram (green; Koldsø et al., 2010) and *R*-fluoxetine (brown; Andersen et al., 2014) from hSERT homology models. LeuBAT labels are shown in normal font, dDAT labels are underlined and hSERT labels are shown in italic. **(E)** Comparison of mazindol binding. Mazindol in LeuBAT crystal structure (white; Wang et al., 2013). Mazindol binding to a hDAT homology model (light purple) and a hSERT homology model (dark purple; Severinsen et al., 2014). hDAT labels are underlined and hSERT labels are italic. The view has been rotate 180 degrees compared to **(A–D)**. **(F)** Comparison of the tricyclic antidepressant binding. Clomipramine (white) in LeuBAT crystal structure (Wang et al., 2013). Imipramine (light orange) and 3-cyano imipramine (dark orange) binding to a hSERT homology model (Sinning et al., 2010). hSERT labels are italic. The view has been rotate 180 degrees compared to **(A–D)**.

Since drug addiction is an enormous burden to society and human health, it is extremely important to understand the molecular mechanism of how these compounds interact with the BATs. Drugs of abuse include inhibitors like cocaine and a class of compounds such as amphetamine, which are able to reverse the direction of transport in BATs by a mechanism that is still not fully understood. This class of molecules is termed “releasers” and includes amphetamine, methamphetamine and some phenyl-piperazine (PP) derived compounds. The binding of PP and an analog has been studies computationally using homology models of hDAT and hSERT (Severinsen et al., 2012). The recently published crystal structure of dDAT included structures that have *D*-amphetamine and (+)-methamphetamine bound (Wang et al., 2015). In Figure 2A an overlay of the two releasers from the crystal structures are displayed along with the position of PP within a hDAT model (Severinsen et al., 2012). As can be observed, there is a pronounced agreement with the position of the releasers within the S1 binding site of DAT. The orientation of PP in hDAT has been observed to be identical to the one observed within hSERT and similar to the orientation of the substrate SERT (Severinsen et al., 2012). The binding of amphetamine described by Beuming et al. (2008) additionally shows this same orientation of amphetamine to a hDAT homology model (Beuming et al., 2008). This could indicate that the substrates and releasers, which are all expected to be transported by the BATs, occupy a similar space within the central binding site and that the orientation is conserved amongst the BATs.

The binding of cocaine has previously been studied through homology modeling both of hDAT (Beuming et al., 2008) and hSERT (Koldsø et al., 2013a). An overlay of the recently published crystal structures of dDAT with cocaine and the cocaine analog RTI-55 (Wang et al., 2015) and the binding model of cocaine in hSERT (Koldsø et al., 2013a) is seen in Figure 2B. Again we observe that the computer models are able to predict the binding of molecules to hSERT and that the orientation and overall position is the same in the model and the crystal structure with a small displacement of the N^+^ group, most likely caused by subtle differences of the phenylalanine within the aromatic lid. The overall location of cocaine in Beuming et al. (2008) is additionally similar to what is also observed in the dDAT crystal structure, further supporting that homology models are indeed able to be predictive of drug binding to proteins. Additionally, the benztropine JHW007 (Desai et al., 2005) has been shown to occupy the same site as cocaine within DAT (Beuming et al., 2008) indicating that benztropines could bind in a similar fashion as cocaine in the dDAT structure.

### Binding of Antidepressants and Mazindol

The binding of the SSRI *S*-citalopram was previously biochemically validated to bind in the central S1 site of hSERT (Koldsø et al., 2010). The recently published dDAT structures by Penmatsa et al. (2015) has revealed that the NRIs nisoxetine and reboxetine bind to the central binding site of dDAT. Figure 2C illustrates that the SSRI *S*-citalopram and the NRIs nisoxetine and reboxetine occupy the same space within the central binding pocket as assessed by comparing the hSERT homology model (Koldsø et al., 2010) and the dDAT crystal structures (Penmatsa et al., 2015). The pharmacology profile of dDAT resembles that of hNET more than hDAT which could suggest that the orientation of NRIs in dDAT is representative of binding to hNET. This further hints to SSRIs and NRIs as possibly binding in a similar fashion in hSERT and hNET respectively.

Numerous SSRIs have been co-crystallized with the LeuBAT structures (Wang et al., 2013). Figure 2D illustrates the overlay between SSRIs bound to LeuBAT and the SSRIs fluoxetine (Andersen et al., 2014) and *S*-citalopram (Koldsø et al., 2010) within a hSERT homology model in addition to nisoxetine bound to dDAT (Penmatsa et al., 2015). Again we see high degree of overlap in spatial orientation of these antidepressants within the central binding site. Some discrepancies are observed between the binding of fluoxetine (Prozac) obtained through modeling (Andersen et al., 2014) and a LeuBAT crystal structure (Wang et al., 2013; Figure 2D). As discussed in details in Andersen et al. (2014) this difference in orientation of the large antidepressant fluoxetine in the model and the engineered LeuBAT can potentially be assigned to the fact that the LeuBAT structure is only partly representing the binding site of hSERT.

Mazindol has been shown to be an anorectic agent (Aeberli et al., 1975) and like cocaine, mazindol binds to all human BATs. The LeuBAT structures published in 2013 revealed the binding of mazindol both to the Δ5 and the Δ6 structures (Wang et al., 2013). In both structures mazindol was found in the same orientation. A comparison of the binding of *R*-mazindol in the Δ6 LeuBAT crystal structure (Wang et al., 2013) with the mode obtained through homology modeling and docking in a hSERT and a hDAT model (Severinsen et al., 2014) is seen in Figure 2E. We observe excellent agreement between the binding modes obtained through the computational studies and the crystal structure of the engineered LeuT protein. Only a small reorientation of the chlorophenyl group is observed between the two crystal structures and the models.

Lastly we have compared the binding mode of TCAs between the LeuBAT structure (Wang et al., 2013) and models obtained through computational studies (Sinning et al., 2010). The TCA CMI was co-crystallized in the Δ13 LeuBAT structure (Wang et al., 2013). The first dDAT structure was also crystallized with a TCA, nortriptyline, and the orientation of this drug is the same in dDAT as CMI in LeuBAT (Penmatsa et al., 2013; Wang et al., 2013). The binding of the TCA imipramine and analogs binding to a hSERT homology model has previously been explored (Andersen et al., 2009; Sarker et al., 2010; Sinning et al., 2010). We observe that the tricyclic ring structure of the antidepressants overlays within the central binding site of hSERT (Sinning et al., 2010) and LeuBAT (Wang et al., 2013). Additionally both the 3-position chlorine substituent in CMI from the crystal structure and 3-cyano imipramine within the hSERT model are orientated toward the aromatic lid of the central binding site (Figure 2F). Again, this illustrates excellent agreement between computational models and subsequent crystal structures as seen both for LeuBAT and dDAT.

## Discussion

We have displayed excellent agreement between the binding modes of numerous types of ligands to homology models of the human BATs and crystal structures of dDAT and the engineered LeuBAT. We have explored and compared the binding of several classes of ligands ranging from the substrates over psychostimulants, including the releasers PP and amphetamines and the inhibitor cocaine, SSRIs, NRIs, to the anorectic drug mazindol and the TCA imipramine and analogs. Binding of several other compounds to BATs has been studied computationally including the anti-abuse drug ibogaine (Koldsø et al., 2013a) and SERT-binding fluorescent drugs (Wilson et al., 2014), however, these still remain to be elucidated by high-resolution structures. The agreement between the crystal structures and computational models illustrate that it is possible to obtain informative and useful models of drug binding to homology models through careful modeling in conjugation with experimental validation. Particularly the structures of dDAT has opened up for a great revenue to use in the exploration of drug binding, while LeuBAT has additionally shown to be illustrative of drug binding modes in the case of SSRIs, mazindol and TCAs.

Interestingly, in the novel structures of dDAT one of the significant conformational changes between an occluded and outward facing state is the rotation of the aromatic lid residues F319 (equivalent to F253 in LeuT, F335 in hSERT, F320 in hDAT, and F317 in hNET). A large number of published homology models of the human BATs (Celik et al., 2008; Koldsø et al., 2010, 2011, 2013a,b; Sinning et al., 2010; Severinsen et al., 2012, 2014; Andersen et al., 2014) have been constructed based on the first crystal structure of LeuT which was found in an outward occluded state (Yamashita et al., 2005). Not surprisingly, the largest difference in the binding site residues between the homology models and the dDAT crystal structures are accordingly the position of the phenylalanine within the aromatic lid (F317 in dDAT; F335 in hSERT, F320 in hDAT, and F317 in hNET) as seen in Figures 1B–D and Figures 2A–D.

We observe that the early models of the human BATs constructed based on the bacterial homolog LeuT are in excellent agreement with the subsequent crystal structures, but importantly to note is that these predictions require careful selection of ligand binding modes combined with the consideration of more than top docking poses. This provides us with great confidence in the ability to use extensive modeling combined with experimental validations to provide initial insight to drug binding to proteins. Although the computational docking models are able to predict the binding of compounds to the BATs, a substantial limitation of the static models is the inability to predict the difference in function between the drugs. To understand the function, non-static methods such as molecular dynamics simulations (Koldsø et al., 2013a; Grouleff et al., 2015) or electron paramagnetic resonance (EPR; Claxton et al., 2010) need to be applied.

The dDAT structure has been seen to possess a pharmacology profile resembling hNET more than dDAT (Penmatsa et al., 2015) which should equally be taken into consideration when interpreting the dDAT structure. Nevertheless, current advances not only in computational models and more recently high resolution information from crystal structures have allowed us to move one step closer to understanding how drugs binding to the pharmaceutically important BATs.

### Conflict of Interest Statement

The authors declare that the research was conducted in the absence of any commercial or financial relationships that could be construed as a potential conflict of interest.
